# Elevated activity of superoxide dismutase in male late-life schizophrenia and its correlation with clinical symptoms and cognitive deficits

**DOI:** 10.1186/s12888-021-03604-5

**Published:** 2021-12-04

**Authors:** Lijuan Huo, Xiaobing Lu, Fengchun Wu, Catherine Chang, Yuping Ning, Xiang Yang Zhang

**Affiliations:** 1 Department of Psychiatry, Affiliated Brain Hospital of Guangzhou Medical University (Guangzhou Huiai Hospital), Guangzhou, 510000 China; 2 Guangdong Engineering Technology Research Center for Translational Medicine of Mental Disorders, Guangzhou, 510000 China; 3 Department of Psychiatry and Behavioral Sciences, The University of Texas Health Science Center at Houston, Houston, TX USA; 4 The First School of Clinical Medicine, Southern Medical University, Guangzhou, China; 5 CAS Key Laboratory of Mental Health, Institute of Psychology, Chinese Academy of Sciences, 16 Lincui Road, Chaoyang District, Beijing, 100101 China

**Keywords:** Geriatric schizophrenia, Oxidative stress, Plasma, Cognition, Negative symptoms

## Abstract

**Background:**

Despite inconsistent findings, accumulative evidence has shown abnormalities of the key antioxidant enzyme, superoxide dismutase (SOD), in patients with schizophrenia. However, few studies explored SOD in late-life schizophrenia (LLS). Our work aimed to investigate changes in SOD activity and the relationship between SOD activity and psychotic symptoms or cognitive deficits in LLS.

**Methods:**

32 geriatric male patients with schizophrenia (age ≥ 60) and 28 age-matched male normal controls were recruited in the study. We assessed cognitive functions with the Repeatable Battery for the Assessment of Neuropsychological Status (RBANS), evaluated the severity of clinical symptoms with the Positive and Negative Syndrome Scale (PANSS), and measured the plasma levels of SOD.

**Results:**

Patients with LLS presented with higher total levels of SOD compared to the controls (81.70 vs. 65.26 U/ml, *p* < .001). Except for the visuospatial index, the cognitive performance was significantly worse on RBANS total and other domain scores in the schizophrenia group than the control group. In the schizophrenia group, SOD levels were positively correlated with subscores of general psychopathology and negative symptoms and total scores of the PANSS (all *p* < .05), and inversely associated with performance in immediate memory, language, and RBANS total scores (all *p* < .05).

**Conclusions:**

Our findings suggest that patients with LLS display disturbances in the antioxidant system, which may underlie the pathological process of cognitive impairments and negative symptoms in the late stage of schizophrenia. Supplementing with antioxidants could be a potential treatment.

## Background

As the global population ages, the number and proportion of older adults with major psychiatric disorders increase considerably. For example, based on the 1% prevalence rate, there are currently10 million patients with late-life schizophrenia (LLS) globally, and there will be over 20 million by 2050. This growing group of LLS patients has been overlooked in scientific research. The present study explores the pathophysiological mechanisms and cognitive deficits in LLS patients.

Schizophrenia is widely recognized as a lifelong psychiatric disorder characterized by three clinical features, i.e., positive symptoms, negative symptoms, and cognitive deficits. Cognitive deficits begin at the earliest stage of the illness, as early as ten years preceding the onset of psychotic symptoms [1–3]. There is increasing evidence that cognitive impairment is one crucial feature leading to poor functional outcome and prognosis [4]. Previous cross-sectional and prospective studies have demonstrated the relationship between cognitive performance and problem-solving skills, social skills acquisition, occupational functioning, and even daily living ability [5, 6]. Cognitive deficits are particularly prevalent among LLS patients, involving global cognition and extensively specific domains, such as executive functions, memory, and language [7, 8]. What’s worse, current first-line antipsychotic medications are ineffective at improving cognitive deficits. The pathophysiological mechanisms underlying cognitive deficits are unclear, resulting in a lack of effective treatment for such deficits.

Oxidative stress is considered part of the pathophysiological mechanism involved in the progression of cognitive dysfunction. Defined as an imbalance between oxidant molecules and the antioxidant defense system, oxidative stress indirectly manifests as altered activities of antioxidants, initiation of lipid peroxidation, and excessive reactive oxygen species (ROS). Oxidative stress has detrimental effects on multiple cellular components, such as proteins, phospholipid membranes, and even DNA, possibly leading to the death of neurons [9]. The oxidative stress hypothesis of aging [10] postulates that physiological aging results from molecular peroxidative damage. For patients with mild cognitive impairment (MCI) and Alzheimer’s disease (AD), the oxidative microenvironment for neurons in the brain undergoes changes in the early stage of illness, followed by oxidative damage [11]. This indicates that abnormal oxidative stress might be a trigger in the onset and progression of AD. Consistently, the contribution of oxidative damage to age-related cognitive dysfunction has been directly verified in studies with animal models. By injecting rats with corticosterone to generate ROS, oxidative damage occurred in the hippocampus, which led to impairment in memory and learning [12]. Treatment with synthetic antioxidants (i.e., superoxide dismutase and catalase mimetics) or vitamin E could significantly relieve oxidative brain load and protect against age-related decline in learning and memory in aged mice [13, 14].

A large body of literature has indicated that the accumulation of oxidative damage underlies the molecular basis of schizophrenia. In both first-episode drug-naïve and chronically medicated patients, abnormalities of antioxidants and lipid peroxidation products have been consistently reported in cerebrospinal fluid, postmortem brain tissues, and peripheral blood (e.g., red blood cells, neutrophils, platelets) [15–17]. Meanwhile, studies have also uncovered significant correlations between the levels of biomarkers of oxidative damage and the severity of psychopathological symptoms or cognitive dysfunction [18, 19].

Taken together, impaired antioxidant defenses systems were found in individuals with both schizophrenia and degenerative diseases and are thus considered as a risk factor for the development of cognitive deficits in these patients. Nevertheless, no studies thus far have examined the levels of antioxidants in LLS patients and the relationships between antioxidants and psychiatric symptoms or cognitive function. Superoxide dismutase (SOD), responsible for detoxifying superoxide radicals, is a dominant enzymatic antioxidant. SOD with two isoforms, the manganese isoform (Mn-SOD) located in mitochondria, and the copper and zinc isoform (CuZn-SOD) in the cytoplasm, plays a vital role in the deactivation of ROS. The present study measured the peripheral levels of SOD as a marker of oxidative stress, aiming to address the following two issues, (1) whether SOD was altered in LLS patients compared to the age-matched control group and (2) whether there was a relationship between SOD levels and clinical symptoms or cognitive functions in older schizophrenia.

## Methods

### Participants

We recruited thirty-two male LLS patients from the public psychiatric hospital, Hui-Long-Guan hospital in Beijing. Patients were diagnosed by two independent and experienced psychiatrists, using structured clinical interviews for DSM-IV (SCID). Eligibility criteria were as follows: (1) males with age older than 60 years, (2) without neurodegenerative diseases, substance abuse, major brain injury, or severe physical diseases, (3) not meeting criteria for other psychiatric disorders except schizophrenia, (4) willingness to provide written informed consent. The average age of patients was 63.63 ± 2.87 years old, and the average age at first onset was 27.06 ± 6.11 years old. All patients took a stable dose of antipsychotics for at least 5 months before participating in this study. Antipsychotic drugs included clozapine (*n* = 12), risperidone (*n* = 11), perphenazine (*n* = 4), pipotiazine (*n* = 2), haloperidol (*n* = 1), and sulpiride (*n* = 2). The average duration of participants taking their current drug was 50.39 ± 50.69 months, and the daily dose was 320.35 ± 159.27 mg (chlorpromazine equivalents).

In addition, twenty-eight healthy male controls were recruited from the local community around the Hui-Long-Guan hospital. A research psychiatrist assesses the mental status and diagnosis of normal controls through structured clinical interviews based on DSM-IV (SCID). The inclusion criteria were comparable to those for patients, except that normal controls were not diagnosed with a personal or family history of psychiatric disorders.

Trained researchers conducted a research interview with each participant to collect detailed social-demographic information and a complete medical and psychiatric history. This study was approved by the Research Ethics Review Board of Beijing Hui-Long-Guan hospital. Before participating in this study, all participants signed a written informed consent form.

### Clinical symptoms and cognitive assessments

The Repeatable Battery for the Assessment of Neuropsychological Status (RBANS) [20] was administered to all participants to evaluate cognitive function. The RBANS consists of 12 neuropsychological tests, which are designed to measure five domains of cognition, including immediate memory, visuospatial/constructional ability, language, attention, and delayed memory. A higher score obtained in the RBANS indicates better cognitive performance. The RBANS takes less than or about 30 min to administer, which has been widely used to detect cognitive impairment in neurological and psychiatric patients.

For each LLS patient, the symptom severity was assessed by two independent psychiatrists using the Positive and Negative Syndrome Scale (PANSS) [21]. As a 30-item semi-structured interview, the PANSS was parsed into three symptom categories related to schizophrenia: positive symptoms, negative symptoms, and general psychopathology. A higher score in PANSS indicates more severe psychopathology. Before the study began, psychiatrists participated in a training course on how to use PANSS. The inter-rater correlation coefficient of the PANSS total score was more than 0.80.

### Determination of SOD activity

We collected 5 ml of blood samples from each participant after an overnight fast, between 7 am and 9 am. Plasma red blood cells were then separated, aliquoted, and stored at − 70 °C until analysis. The activity of total SOD and two SOD isoenzymes, Mn-SOD and CuZn-SOD, were then analyzed with commercially available assays.

The assay of plasma SOD activities was performed according to the manufacturers’ instructions by measuring the inhibition of superoxide-induced formation of nitrite from hydroxylamine using spectrophotometric determination [22]. The activity was expressed as Units per milliliter plasma (U/ml). One unit of total SOD is defined as the amount of SOD required to inhibit 50% of nitrite formation. The activity of Mn-SOD was evaluated after inhibiting CuZn-SOD activity by adding cyanide (1 mM), and the CuZn-SOD activity was determined by subtracting Mn-SOD activity from total SOD activity. The inter-assay and intra-assay coefficient of variation for SOD activity was 4 and 3%, respectively. The samples from patients and controls were collected simultaneously, stored for a similar time, and then assayed together by a research assistant who was blind to the clinical status of the participants.

### Statistical analysis

There were no outliers (> = ± 3SD) in SOD activity, clinical symptoms, and cognition. The majority of continuous variables were normally distributed (Shapiro–Wilk test). Differences in demographic data between the study groups were tested by independent *t*-tests for continuous variables and chi-squared tests for categorical variables. SOD activity and cognitive performance of the control and LLS patient groups were compared by one-way analyses of covariance (ANCOVA), with age, years of education, marital status, and smoking status as covariates. Pearson’s correlation coefficients were then employed to examine the relationship between SOD activity, clinical symptoms, and cognitive performance in each group. Finally, stepwise regression analyses were performed to further explore the relationship between SOD activity, demographic and clinical variables, and cognitive performance. Dependent variables were RBANS total or index score and independent variables included SOD activity, age, years of education, smoking status, marital status, types and dosage of antipsychotics, and PANSS scores. We used the Bonferroni correction for multiple tests in all correlation analyses.

## Results

### Demographic characteristics

Socio-demographical and clinical features of LLS patients and healthy elderly controls are displayed in Table 1. The two groups did not differ significantly in age and BMI but differed in years of education, marital status, and the number of smokers. Accordingly, age, years of education, marital status, and smoking status were adjusted in the following analyses.

**Table 1:** Characteristics of late-life schizophrenia (LLS) and healthy older adults

	LLS (*n* = 32)	Controls (*n* = 28)	t or χ ^2^	*p*
Age (years)	63.63 ± 2.87	63.96 ± 3.16	−.44	.66
Education (years)	10.0 ± 2.81	7.89 ± 3.48	2.56	.013
BMI (kg/m^2^)	23.86 ± 6.55	26.08 ± 2.90	−1.55	.23
Marital status			26.88	<.001
Single	50%	0		
Married	25%	89.3%		
Divorced or widowed	25%	10.7%		
Cigarette smoking	68.8%	0	30.40	<.001
Age of onset (years)	27.06 ± 6.11	–		
Antipsychotic		–		
Atypical	78.1%	–		
Typical	21.9%	–		
Antipsychotic dose (mg/day, chlorpromazine equivalents)	320.35 ± 159.27	–		
**PANSS**		–		
Total score	65.87 ± 10.46	–		
Positive symptoms subscore	13.71 ± 5.15	–		
Negative symptoms subscore	24.16 ± 5.98	–		
General psychopathology subscore	28 ± 4.66	–		

*PANSS* the Positive and Negative Syndrome Scale

### Plasma SOD activity in LLS and healthy older controls

We found significant group differences in the levels of total SOD (*F* = 50.84, *p* < .001) and CuZn-SOD (*F* = 10.75, *p* = .002). Accordingly, higher levels of total SOD (81.70 vs. 65.26 U/ml) and CuZn-SOD (59.25 vs. 47.11 U/ml) were observed in the LLS group. The difference remained significant in further ANCOVA analysis using age, years of education, marriage status, and smoking status as covariates. The levels of another isoenzyme of SOD, Mn-SOD, were higher in the LLS group than in the control group, but the difference was not statistically significant. The specific levels of these markers and statistic values are summarized in Table 2.

**Table 2:** Oxidative stress parameters in late-life schizophrenia (LLS) versus healthy older controls

Variables	LLS (*n* = 32)	Controls (*n* = 28)	*F*	*p*	Adjusted F^*^	Adjusted p^*^
SOD	81.70 ± 9.83	65.26 ± 7.73	50.84	<.001	31.43	<.001
CuZn-SOD	59.25 ± 15.54	47.11 ± 12.73	10.75	.002	8.12	.006
Mn-SOD	22.45 ± 11.75	18.14 ± 13.25	1.78	.187	.56	.46

*Adjusted values were calculated with age, marital status, education, and smoking as covariates

*SOD* superoxide dismutase; *CuZn-SOD* the manganese (Mn) isoform SOD; *Mn-SOD* the copper and zinc (CuZn) isoforms SOD

### Correlation between SOD activity and psychotic symptoms

**Fig. 1:**
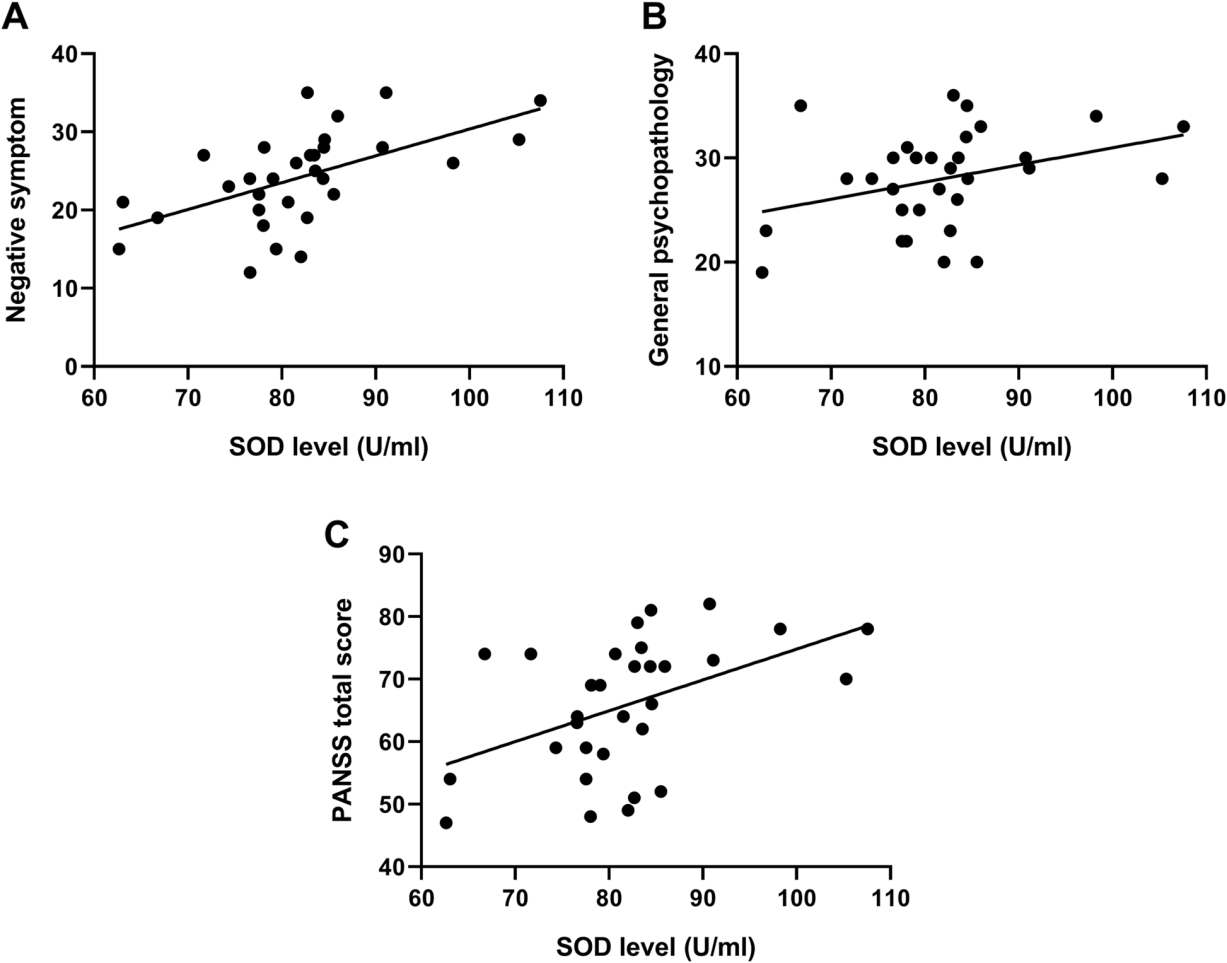
Positive association between the superoxide dismutase (SOD) levels with (**A**) negative symptoms subscore (*r* = .57, *p* = .001), (**B**) general psychopathology subscore (*r* = .35, *p* = .054), and (**C**) total sore of PANSS (*r* = .47, *p* = .008) in patients with late-life schizophrenia. ^*^uncorrected *p* < .05

In LLS patients, plasma SOD activities were positively related to the severity of clinical symptoms, except for positive symptoms, measured with the PANSS (Fig. 1). In other words, the higher levels of total SOD were associated with the higher scores obtained in negative symptom (*r* = .57, *p* = .001), general psychopathology (*r* = .35, *p* = .054), and the total PANSS (*r* = .47, *p* = .008). Following the Bonferroni correction, the correlation between the levels of total SOD and scores of negative symptoms and the total scores of the PANSS remained significant.

### Cognitive performance of LLS and healthy older controls

Table 3 shows cognitive characteristics of LLS patients and healthy controls. There were significant differences in all domains of cognition, except for the performance of visuospatial/constructive ability. LLS patients showed poorer performance on immediate memory, attention, language, delayed memory, and total scores of RBANS in comparison with healthy elderly controls (all *p* < .05). After adding age, education, marital status, and smoking status as covariates in the further ANCOVA, the group differences remained significant on performance in immediate memory, delayed memory, language, as well as total scores of RBANS (all *p* < .05), and a marginal significance on performance in attention (*p* = .074).

**Table 3:** Total and each index scores on the RBANS in late-life schizophrenia (LLS) versus healthy older controls

Index	LLS (*n* = 32)	Controls (*n* = 28)	*F*	*p*	Adjusted F^*^	Adjusted p^*^
Immediate memory	57.53 ± 13.20	73.48 ± 17.24	16.18	<.001	13.70	.001
Attention	75.66 ± 13.31	88.26 ± 21.38	7.63	.008	3.34	.074
Language	86.50 ± 12.83	94.26 ± 9.04	6.96	.011	12.76	.001
Visuospatial	82.75 ± 15.27	76.52 ± 12.31	2.90	.094	1.55	.219
Delayed memory	66.75 ± 19.37	86.0 ± 17.12	16.07	<.001	24.73	<.001
Total scale	67.16 ± 12.06	79.11 ± 14.52	11.94	.001	13.94	<.001

*Adjusted values were calculated with age, marital status, education, and smoking as covariates. *RBANS* the Repeatable Battery for the Assessment of Neuropsychological Status

For the LLS group, cognitive performance was associated with psychotic symptoms. Specifically, performance of immediate memory was negatively related to the subscores of negative symptom (*r* = −.38, *p* = .036), general psychopathology (*r* = −.45, *p* = .011), and the total scores of the PANSS (*r* = −.39, *p* = .031). Performance of language was inversely associated with the severity of negative symptom (*r* = −.49, *p* = .005) and general psychopathology (*r* = −.38, *p* = .035). Attention ability was also negatively associated with the sub-scores of general psychopathology (*r* = −.37, *p* = .04). The total scores of RBANS were negatively related to the subscores of general psychopathology (*r* = −.42, *p* = .019). However, the significance did not survive the multiple comparison correction.

### Correlation between SOD activity and cognitive performance

**Fig. 2:**
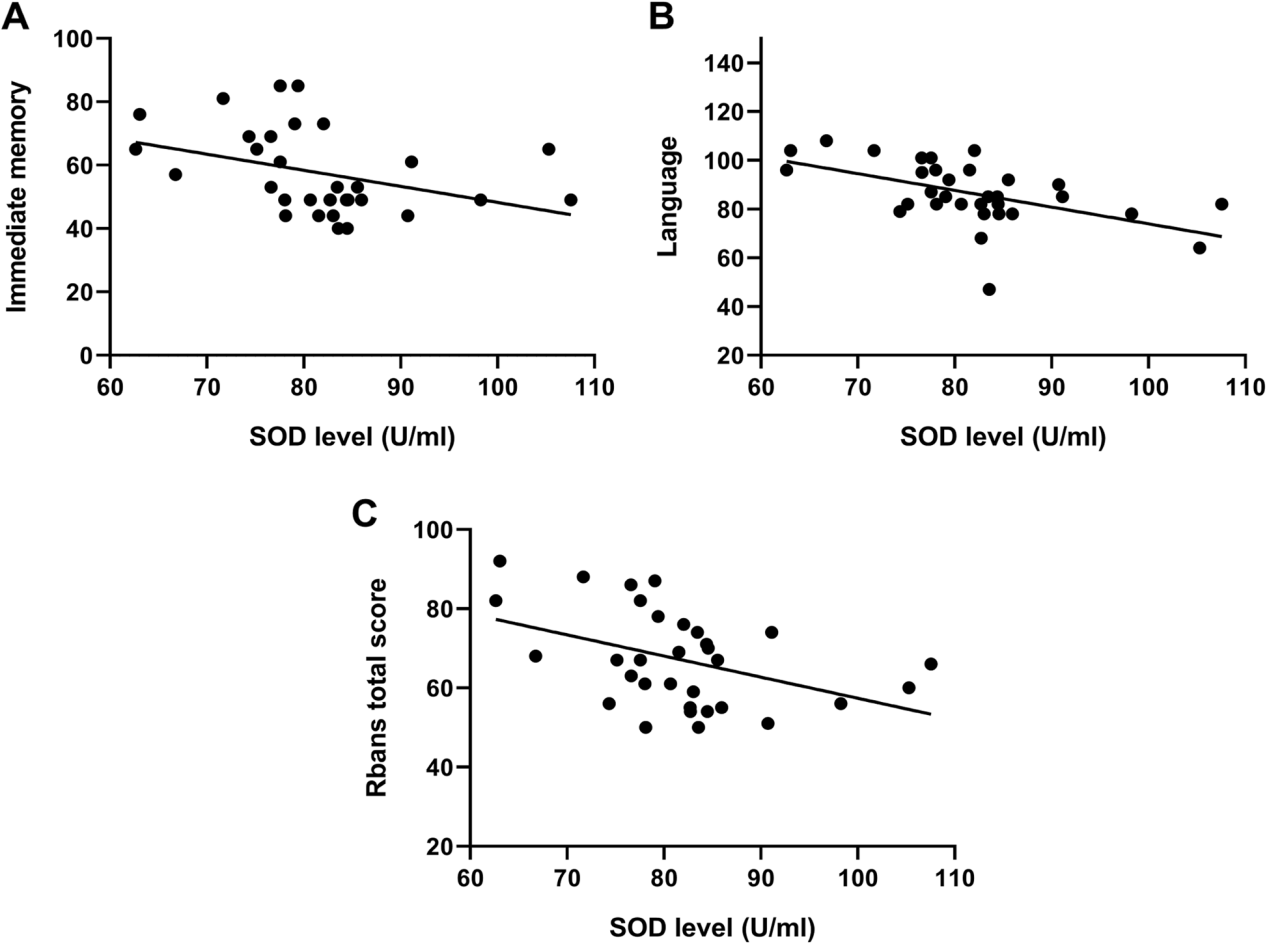
Negative association between the superoxide dismutase (SOD) levels with (**A**) immediate memory subscore (*r* = −.38, *p* = .033), (**B**) language subscore (*r* = −.53, *p* = .002), and (**C**) total sore of RBANS (*r* = −.44, *p* = .013) in patients with late-life schizophrenia. ^*^uncorrected *p* < .05

First, the relationship between SOD activity and cognitive performance in each group was calculated. Focusing on the LLS group, the total SOD activities were negatively associated with performance of immediate memory (*r* = −.38, *p* = .033), language (*r* = −.53, *p* = .002) and the total score of RBANS (*r* = −.44, *p* = .013, seeing Fig. 2 for details). After Bonferroni correction, the correlation between SOD levels and language ability remained significant (*p* < .05/5), whereas SOD levels were not significantly related to other cognitive indexes (all *p* > .05). The levels of the other two SOD isoenzymes, CuZn-SOD and Mn-SOD, were not found to be associated with any domains of cognitive function (all *p* > .05).

Furthermore, to examine the correlation between total SOD activity, demographic and clinical variables, medication, and cognitive performance, multiple linear regression analyses were performed. In the stepwise regression model using the RBANS total scores as the dependent variable, total SOD levels (*β* = −.36, *t* = − 2.45, *p* = .022) together with years of education (*β* = .44, *t* = 2.98, *p* = .006) and marital status (*β* = −.32, *t* = − 2.20, *p* = .037) were correlated with the RBANS total scores, together contributing 47.3% explanation for variation. In the model using the language index as the dependent variable, only total SOD levels (*β* = −.54, *t* = − 3.33, *p* = .003) were significantly linked with language, with 29.1% R square. In stepwise models with the performance of other cognitive domains as the dependent variable, total SOD levels were not allowed to enter the models.

In the control group, there were no significant associations between the plasma levels of these oxidative stress markers and cognitive performance.

## Discussion

To our best knowledge, this is the first study investigating the levels of an oxidative stress marker and its relationship with clinical symptoms and cognitive function in the late stage of male schizophrenia. The major results revealed that (1) the plasma levels of total SOD and the CuZn-SOD, but not Mn-SOD, were significantly elevated in patients with LLS in comparison with the normal control group; (2) LLS patients had lower RBANS scores than the normal controls on cognitive performance in all domains except for the visuospatial and attention ability; (3) the levels of total SOD were positively correlated with the severity of negative symptoms and general psychopathology and the total PANSS score in LLS patients; (4) the levels of total SOD were reversely correlated with immediate memory, language, and the global composite score of RBANS in LLS patients.

The first major finding is that peripheral levels of total SOD were substantially elevated in LLS patients. A possible explanation for the increase of SOD is the compensatory physical reaction to the overproduction of ROS. In other words, increased ROS may trigger the antioxidant defense system as a self-protection mechanism. This kind of increased effort attempting to overcome oxidative damage is consistent with the compensatory response in the schizophrenic brain. Revealed in neuroimaging research, individuals with schizophrenia have shown hyperactivation in prefrontal regions facilitating cognitive performance [23]. In fact, elevated activity of antioxidant enzymes is a common finding in individuals susceptible to oxidative damage, such as patients with neurodegenerative disease and other neuropsychiatric disorders [24, 25], possibly to maintain antioxidant capacity against high levels of ROS. However, increased lipid peroxidation with or without accompanying changes of antioxidant enzymes in schizophrenia have been extensively reported in multiple previous studies [16, 26–28], suggesting that the compensatory response is ineffective.

Currently, the data concerning the status of SOD in adult schizophrenia across existing studies is contradictory. A number of previous studies paralleled with our results report higher activities of SOD in adult patients with schizophrenia [25, 29–32], while other studies demonstrated decreased or unchanged activities of SOD [33–37]. We speculate that due to the great heterogeneity of patients across studies (e.g., age, smoking status, duration and dose of medication, disease severity), patients underwent different stages of oxidative stress and showed various activities of SOD. For instance, even short-term antipsychotic therapy altered SOD activities in medicated patients compared with controls [34, 38]. Regarding our present study, one strength was the high homogeneity of patients. Our valuable results describe the status of SOD in male inpatients in the late stage of schizophrenia with previous long-term medication.

Another meaningful finding was the close relationship between SOD activity and the clinical features of geriatric schizophrenia, especially negative symptoms and general psychopathology. Consistently, negative symptoms increased and dominated in the course of schizophrenia as patients aged [39], which could possibly be explained as the increase in oxidative stress and damage that occurs in aging. In previous studies, the levels of antioxidants were also related to negative symptoms [40, 41] and positive symptoms [42]. These results implied that accumulating disturbances in the antioxidant system might contribute to the severity and characteristics of schizophrenia symptoms, especially in geriatric patients who suffer more oxidative damage.

As a cross-sectional study, we could not directly determine whether alterations in antioxidant capacity were a cause or outcome of the psychopathology in schizophrenia. Interestingly, intervention studies seem to support the former explanation. Dietary supplementation of antioxidants, such as vitamin C, vitamin E, and alpha-lipoic acid, resulted in restoring oxidative neuropathology in patients with schizophrenia, along with amelioration or remission in psychopathological symptoms of schizophrenia [38, 43].

Furthermore, this is the first study to reveal that in patients with LLS, peripheric SOD levels were independently associated with cognition, including immediate memory, language, and general cognitive function, in accordance with previous reports in adult schizophrenia [37, 42]. An increase in SOD levels and oxidized DNA has also been detected in the frontal cortex and hippocampus of the postmortem brain [44, 45]. Following continuous oxidative stress, excessive ROS invariably attacks many molecules, including lipids, proteins, and DNA, eventually leading to cellular degeneration and apoptosis. Recently, Maas and his colleagues proposed the redox-induced prefrontal OPC (oligodendrocytes precursor cells)-dysfunction hypothesis to explain cognitive deficits in schizophrenia [46]. The hypothesis suggests that oxidative stress prevents OPC from proliferating and maturing, thus resulting in hypomyelination and disruption of connectivity in the prefrontal cortex, which is responsible for a variety of higher-level cognitive functions. The above findings provide further evidence that oxidative stress in specific neuroanatomical distribution, which is also reflected by alterations of SOD and other antioxidants in the blood, is involved in the compromised cognitive processes of schizophrenia.

It should be noted that common theories, the neurodevelopmental or neurodegenerative models, postulate that complex interactions between environmental events, genetics factors, and neurochemical pathways contribute to the development of schizophrenia and its cognitive deficits and psychotic symptoms [47]. For example, our previous results uncovered that the interaction between oxidative damage and neurotrophin (BDNF) dysfunction plays a role in the pathological process of this disease [19]. Therefore, further studies will be needed to reveal the extent that oxidative stress contributes to the etiology of schizophrenia and how it interacts with other risk factors.

The present study has some limitations. First, due to the relatively small sample size in this study, our results were preliminary and need to be further verified in an expanded sample of LLS patients. Second, due to the shortage of participants and other limited conditions, we only recruited 28 normal elderly controls. The unmatched number of the cases and controls (32 vs. 28) may lead to deviations in the statistical analysis. Third, although RBANS is a widely used screening instrument in cognitive assessment, it cannot assess all of cognitive domains that may be impaired in patients, such as executive function. In the future studies, we should further recruit more cognitive tests to investigate the relationship between oxidative stress and cognitive impairment. Fourth, in this study, since all patients went through long-term hospitalization, there were many relevant clinical variables that may affect their SOD and cognition, such as food habits, unhealthy lifestyle, and physical exercise, although some factors were controlled in these inpatient individuals. Unfortunately, we did not collect all relevant variables, which may affect the results of SOD levels and cognitive performance. Fifth, we do not determine with certainty whether blood SOD levels reflect the oxidative status in the central nervous system (CNS). Fortunately, accumulative data has shown close correlations between biomarkers of oxidative stress in the peripheric tissues and that in the CNS (cerebrospinal fluid or postmortem) [48]. Sixth, only one specific antioxidant enzyme, SOD, and its isoforms were selected and measured as the biomarker of oxidative stress. Although SOD is a crucial enzyme in the antioxidant system, it might exert influence together with other enzymes and thus might not represent the general antioxidant capacity. Synthetic index, such as total antioxidant status, measured as a ferric reducing antioxidant, is potentially a more accurate representation of the antioxidant status. Last but not least, we only recruited male geriatric patients with schizophrenia in this study for two reasons. First, previous studies have found sex differences in clinical severity, cognitive impairment, and oxidative stress status [49–51]. As a result, we only recruited male patients to ensure sample consistency. Second, in the psychiatric hospitals where we collect data, male elderly patients were more than females. However, our findings may not be generalized to female patients without further evidence.

## Conclusions

In conclusion, the current study demonstrates that in male patients with LLS, SOD activity was significantly increased compared to matched normal controls. Total SOD levels were related with general psychopathology, negative symptoms, and the total scores of PANSS. Moreover, total SOD levels also correlated with a variety of cognitive domains. These findings have important implications for the diagnosis and treatment of schizophrenia in the late stage. First, peripheral measurement of antioxidant enzymes could serve as a potential biomarker predicting the clinical course of schizophrenia. What’s more, our findings support that the supplement of antioxidants may have therapeutic applications for schizophrenia. Since conventional pharmacotherapy comes with severe side-effects and is limited in reducing negative symptoms and enhancing cognitive function, developing potential pharmacological alternatives would greatly benefit patients with LLS.

## Data Availability

The data will be available and provide by the corresponding authors if necessary.

## Abbreviations

SOD: Superoxide dismutase
LLS: Late-life schizophrenia
RBANS: The repeatable battery for the assessment of neuropsychological status
PANSS: The positive and negative syndrome scale
MCI: Mild cognitive impairment
AD: Alzheimer’s disease
DSM-IV: Diagnostic and statistical manual of mental disorders-IV
OPC: Oligodendrocytes precursor cells
ROS: Reactive oxygen species
CNS: Central nervous system
